# COVID-19 Pediatric Follow-Up: Respiratory Long COVID-Associated Comorbidities and Lung Ultrasound Alterations in a Cohort of Italian Children

**DOI:** 10.3390/children11020166

**Published:** 2024-01-27

**Authors:** Cristiana Indolfi, Angela Klain, Giulio Dinardo, Elisabetta D’Addio, Serena Ferrara, Fabio Decimo, Giorgio Ciprandi, Maria Angela Tosca, Michele Miraglia del Giudice

**Affiliations:** 1 Department of Woman, Child and General and Specialized Surgery, University of Campania Luigi Vanvitelli, 80138 Naples, Italy; cristiana.indolfi@policliniconapoli.it (C.I.); giulio.dinardo@studenti.unicampania.it (G.D.); elisabetta.daddio1@studenti.unicampania.it (E.D.); serena.ferrara@studenti.unicampania.it (S.F.); fabio.decimo@unicampania.it (F.D.); michele.miraglia@unicampania.it (M.M.d.G.); 2 Department of Medicine, Allergy Clinic, Casa di Cura Villa Montallegro, 16145 Genoa, Italy; gio.cip@libero.it; 3 Pediatric Allergy Center, Istituto Giannina Gaslini, 16147 Genoa, Italy; mariangelatosca@gaslini.org

**Keywords:** children, COVID-19, follow-up, long COVID, comorbidities, allergy, asthma, lung ultrasound

## Abstract

In children, the factors that influence COVID-19 disease and its medium- and long-term effects are little known. Our investigation sought to evaluate the presence of comorbidity factors associated with respiratory long COVID manifestations in children and to study ultrasound abnormalities following SARS-CoV-2 infection. Children, who arrived at the ‘Respiratory Diseases of Pediatric Interest Unit’ at the Department of Woman, Child, and General and Specialized Surgery of the University of Campania ‘Luigi Vanvitelli’, were selected during the timeframe from September 2021 to October 2022. The children were diagnosed with a SARS-CoV-2 infection that occurred at least one month before the visit. All patients followed a COVID-19 follow-up protocol, developed by the Italian Society of Pediatric Respiratory Diseases (SIMRI), which included: collection of data regarding SARS-CoV-2 illness and history of known respiratory and allergic diseases; physical examination; BMI assessment; baseline spirometry and after bronchodilation test; six-minute walking test; and lung ultrasound (LUS). In a cohort of 104 participants with respiratory long COVID symptoms (64.7% male, average age 8.92 years), 46.1% had fever with other symptoms, and 1% required hospitalization. BMI analysis showed 58.4% of the cohort was overweight. The LUS was positive in 27.0% of cases. A significant BMI association was observed with COVID-19 symptoms and LUS score (*p*-value < 0.05). No associations were found with asthma or atopy.

## 1. Introduction

The worldwide population has experienced the significant impacts of the COVID-19 pandemic and the corresponding health-related interventions on individuals’ lives. The SARS-CoV-2 outbreak has had adverse psychological and health consequences, particularly for young children and teenagers, who experienced the rigor of the lockdown measures undertaken to limit the spread of the virus more significantly than adults [1]. In most cases, in children, COVID-19 seems to manifest as a mild illness, resembling those seen in seasonal respiratory viral infections [2]. The most common presenting complaints are generalized symptoms like fever, cough, headache, diarrhea, and vomiting, and less frequently, anosmia and ageusia [3,4,5,6]. In Europe, throughout the pandemic years of 2021–2022, the hospitalization rate for COVID-19 among all children who tested positive for SARS-CoV-2 ranged from 7.13 to 35.9 per 10,000 children [7,8,9]. While the initial stage of the illness often shows no or mild symptoms, certain children who have had COVID-19 might experience lingering effects even after recovery. These persistent symptoms may encompass psychological, cognitive, and mood issues, such as behavioral and sleep disorders, challenges with concentration and memory, as well as respiratory signs like cough, dyspnea, and chest pain [10,11,12,13]. More recent research on ‘long COVID’ suggest a prevalence of 25.24% in children [14]. In Italy, Morello et al. conducted a study involving more than a thousand of children with SARS-CoV-2 infection, symptomatic or not. They were evaluated at intervals of 3, 6, 12, and 18 months post-infection. At the 3 month mark, 23% of cases were diagnosed with long COVID. Among these, approximately 50% continued to exhibit symptoms at 6 months, around 13% at 12 months, and 5% after a year and a half [15]. Several descriptions or definitions of long COVID have been reported by international societies [16,17,18]. The initial definition came from the World Health Organization (WHO). The WHO’s definition of long COVID or post-COVID-19 conditions is ‘Symptoms occurring at least three months after probable or confirmed SARS-CoV-2 infection. Symptoms must last for at least two months and cannot be explained by an alternative diagnosis. Symptoms may be new onset following recovery from acute SARS-CoV-2 infection or persist from the initial illness. Symptoms may fluctuate or relapse’. The challenge in defining and gauging long COVID has been and remains the primary factor restricting current understanding of this argument [19]. In 2022, an Italian intersociety consensus stated that children exhibiting symptoms of any organic issue consistent with long COVID must undergo a comprehensive evaluation, potentially involving clinical, laboratory, and/or radiological investigations [20]. 

### COVID-19 and Atopic Diseases in Children

A complete understanding of how asthma affects the vulnerability and severity of COVID-19 is yet to be elucidated. Initially, asthmatic and allergic individuals were believed to face a higher risk of developing severe SARS-CoV-2 illness due to their compromised immune responses and heightened susceptibility to respiratory exacerbations when exposed to respiratory viruses. However, numerous studies conducted during the SARS-CoV-2 pandemic have underscored a diminished likelihood of serious COVID-19 in patients suffering from asthma and allergies [11,21]. In line with the systematic review conducted by Choi et al., asthma did not emerge as a risk factor for severe COVID-19 in children [22,23]. In an Italian survey comprising about 200 children, Tosca et al. observed no notable difference in hospitalization rates, pneumonia prevalence, and the prevalence of oxygen therapy between allergic and non-allergic patients concerning COVID-19 disease [24]. In a survey proposed by the European Respiratory Society (ERS), only 5% of 945 SARS-CoV-2-infected children were asthmatic [25]. Furthermore, according to the Italian Pediatric Society of Allergy and Immunology (SIAIP), if effectively managed, allergic rhinoconjunctivitis and asthma do not pose as risk factors for susceptibility to SARS-CoV-2 [26,27]. Asthmatic children experienced fewer exacerbations during the pandemic due to the enforcement of lockdown measures, including the closure of schools, which limited the transmission of viral diseases and minimized the likelihood of exposure to asthma triggers, such as viral infections, outdoor allergens, pollution, and physical activities [28,29,30].

A paradoxical phenomenon that has been reported is that individuals with asthma and allergies have encountered COVID-19 with a lower frequency and reduced severity than their counterparts [31,32,33]. Certainly, a significant number of children with asthma were already undergoing treatment with corticosteroids, which resulted in being effective in severe cases of COVID-19 [34]. However, there would need to be additional evidence to support this hypothesis. Atopy and type 2 inflammation have been associated with a decreased expression of ACE2 in airway epithelial cells and thus lower susceptibility to SARS-CoV-2 [35]. Furthermore, hypereosinophilia, which is characteristic of allergic diseases and type-2 asthma, may act as a protective factor in children with COVID-19 [21,36,37]. Several studies have observed absolute eosinophil counts below the normal range in COVID-19 patients upon admission [38,39].

While most systematic reviews and meta-analyses seem to dismiss the connection between asthma, atopy, and COVID-19, the link between asthmatic/allergic diseases and long COVID remains not entirely understood. According to many studies, allergic diseases represent a risk factor for persistent COVID-19 symptoms in children [40,41,42]. On the other hand, in the study by Fernández-de-Las-Peñas et al., the presence of long-term post-COVID-19 symptoms was similar between patients with and without pre-existing asthma, suggesting that asthma seems not to be a risk factor for more severe long-term post-COVID-19 symptoms but rather could be a “protective” factor for more severe long-term post-COVID-19 symptoms [43]. In the latest meta-analysis by Zheng et al., factors such as older age, being female, obesity, compromised physical or mental health, pre-existing respiratory conditions, hospitalization, and experiencing severe acute COVID-19 were identified as being more prevalent among the pediatric population with long COVID [44].

In our investigation, we assessed the role of comorbidities, including asthma and atopic disease, in pediatric patients experiencing long COVID respiratory symptoms and their correlation with lung abnormalities detected through lung ultrasound (LUS).

## 2. Materials and Methods

Our study is an observational, retrospective, single-center study. We enrolled pediatric patients who arrived at the ‘Respiratory Diseases of Pediatric Interest Unit’ at the Department of Woman, Child, and General and Specialized Surgery of the University of Campania ‘Luigi Vanvitelli’. Our department established an outpatient service catering to pediatric patients with a SARS-CoV-2 infection with persisting respiratory symptoms (cough, dyspnea, wheezing, etc.). Within this initiative, we sequentially included pediatric patients between September 2021 and October 2022. Patients were required to fulfill the following eligibility criteria: (1) age ranging from 3 to 18 years; (2) confirmed diagnosis of COVID-19 through a nasopharyngeal swab; (3) occurrence of SARS-CoV-2 infection at least one month prior to the visit; (4) respiratory symptoms (lingering or new onset cough and/or dyspnea) that lasted more than 4 weeks from the diagnosis, consistent with long COVID manifestations.

Informed consent was acquired from the parent or caregiver for each patient. The study received approval from the ethics committee of the University of Campania ‘Luigi Vanvitelli’, ensuring compliance with the Declaration of Helsinki (register number 0029465).

All patients followed a COVID-19 follow-up protocol, developed by the Italian Society of Pediatric Respiratory Diseases (SIMRI), which included: (a) collection of data regarding SARS-CoV-2 infection and history of known respiratory and allergic diseases; (b) physical examination; (c) BMI assessment; (d) baseline spirometry and after bronchodilation test; (e) six-minute walking test; (f) LUS [45].

### 2.1. Assessment Tools

**Figure 1:**
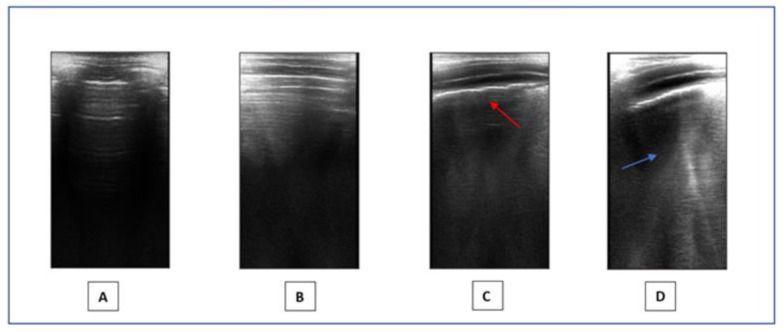
LUS. (**A**,**B**)**:** Normal images. (**C**,**D**): Pathological pattern: irregularities of the pleural line (red arrow) and presence of coalescent B-lines (blue arrow).

For the collection of the children’s parameters and history, we used a patient card (Supplementary Materials, File S1), which included:

a. Medical Questionnaire: Our initial step involved collecting data on the acute disease caused by SARS-CoV-2 by filling out a questionnaire provided to parents. This encompassed confirming SARS-CoV-2 diagnosis and recovery through nasopharyngeal swabs, documenting symptomatology (asymptomatic, cough, fever, anosmia, ageusia, muscular pains, etc.), duration of illness (less than or greater than 15 days), administered treatments (i.e., paracetamol, corticosteroids, non-steroidal anti-inflammatory drugs, oxygen supplementation, etc.), and the necessity of hospitalization. Additionally, we gathered information about the patient’s demographic and medical history concerning known allergic and respiratory conditions and previous allergic sensibilizations confirmed by validated tests (skin prick test, IgE specific count).
b. Physical examination and BMI assessment: We visited the children, collected data from their respiratory physical examinations and recorded their weight and height to calculate their BMI. As per the Expert Committee Recommendations, being underweight is defined as having a BMI below the 5th percentile, while overweight is categorized between the 85th and less than the 95th percentile. Obesity is characterized by a BMI at or exceeding the 95th percentile for children and teenagers [46].
c. Baseline spirometry and after bronchodilation test: Spirometry plays an important role in evaluating pulmonary health and detecting early signs of respiratory disorders in children. Spirometry provides a comprehensive analysis of lung function parameters, including forced vital capacity (FVC), forced expiratory volume in one second (FEV1), FEV1/FVC ratio, and forced expiratory flow between 25% and 75% (FEF 25–75%) of FVC. These measures aid in diagnosing various respiratory conditions including conditions like asthma and restrictive lung disorders in pediatric patients [47]. In our study, spirometry was used to measure FVC, FEV1, FEF 25–75%, and the FEV1/FVC ratio in patients aged 6 years old or younger if capable of performing it. Spirometry was performed according to ERS consensus guidelines [48]. An FEV1 < 80% and FVC < 80% of predicted values were considered abnormal, while FEF 25–75% values were considered abnormal when they were <70% of those predicted. A rise in FEV1 of ≥12% and/or ≥200 mL after inhaling 400 µg of a short-acting β2-agonist (salbutamol) was considered a significant bronchodilator response [49].
d. Six-minute walking test: the six-minute walking test is a simple, practical test used to measure the functional exercise capacity. During the examination, participants are directed to walk for the maximum distance achievable within six minutes along a long, flat, straight, enclosed corridor [50,51,52]. In our study, for technical reasons, this test was performed on a treadmill. The goal for the patients was to walk at their own pace in six minutes. They could rest at any time during the test. During the test, heart rate (HR) and oxygen saturation (SpO2) were continuously monitored, using a finger pulse oximeter. The onset of symptoms such as dyspnea and cough has also been documented.
e. Lung ultrasound: LUS is a non-invasive method that provides a real-time image of lung structures, enabling doctors to identify early alterations in lung function. The radiation-free approach and portability of ultrasound make it an attractive choice in children, helping to limit exposure to X-rays. LUS has gradually expanded its use to encompass various pediatric applications, including all types of pneumonia, pulmonary embolism, and typical chest and lung diseases in childhood [53]. Lung semiotics consists of artifacts originating from the air/tissue interface and authentic images, like effusions and consolidations [54]. Non-pathological ultrasound images exhibit A-lines and a consistently thin pleural line. Conversely, abnormal images are identified by the observation of three or more B-lines between two ribs in a single scan with the disappearance of A-lines, indicating subpleural interstitial edema, up to a ‘white lung’ picture, consolidations, an irregular or thickened pleural line, and pleural effusion (Figure 1). LUS is a very effective and sensible method for assessing the presence of small pleural effusion. Furthermore, in the diagnosis of childhood pneumonia, lung ultrasound (LUS) is considered as an imaging alternative to computed tomography (CT) scans, as LUS findings demonstrate a significant correlation with those observed in chest CT scans [55]. Nonspecific abnormalities, such as the presence of multiple bilateral B-lines, indicating a reduction in air content, thickening of the pleural line with associated abnormalities, and peripheral consolidation, may be observed on the LUS scans of children with COVID-19 pneumonia [56]. Since the early stages of the pandemic, LUS has demonstrated to be a helpful tool to assess lung conditions, monitor any changes over time, and guide therapeutic decisions in both adults and children [57,58,59,60,61]. The LUS in this study was conducted using a linear probe. We adhered to a standardized approach regarding acquisition protocol, as previously outlined by Volpicelli et al. [62]. We examined a total of 12 thoracic areas: 2 anterior, 2 lateral, and 2 posterior areas on each side. Based on the severity of the findings, a numerical score between 0 and 3 was assigned: 0 = normal; 1 = irregular pleural line with less than three B-lines between two ribs in a single scan; 2 = irregularities of the pleural line with more than three B-lines between two ribs in a single scan; 3 = areas of consolidations or ‘white lung’. Each LUS was performed by the same doctor, Dr. A.K., who is a pediatrician and a certified expert operator in pediatric lung ultrasound.

### 2.2. Statistical Analysis

The demographic characteristics of the patients were delineated through descriptive statistics and presented as percentages. Subsequently, we compared variables such as the presence of asthma, gender, BMI values, and vitamin D levels with the results obtained from the spirometry, lung ultrasound, and COVID-19 symptom analyses using the chi-square test. A significance threshold of *p* < 0.05 was established to ascertain statistical significance.

## 3. Results

We recruited 104 patients, 64.7% of which were male. The mean age was 8.92 years; 17.6% of patients had asymptomatic SARS-CoV-2 illness, 35.3% solely experienced fever, 46.1% had fever along with another symptom/sign, and 1% required hospitalization with oxygen therapy. Regarding the duration of COVID-19 disease, 55.9% were affected for less than 15 days, while 44.1% had it for a duration longer than 15 days. As for BMI, 20.8% possessed a BMI falling within the 85th and 95th percentiles, while 37.6% had a BMI exceeding the 95th percentile. In general, 58.4% of children had a BMI > 85th, indicating overweight. Moreover, 28.9% of patients suffered from allergic rhinitis, 46.1% had asthma, and 56.9% reported an allergic sensitization. Chest auscultation was normal in all children, except for 13 patients who presented pathological respiratory sounds (rattles, crackles, and wheezes). During the spirometry, only two children exhibited an FEV1/FVC ratio below 80%, which was reversible after the bronchodilation test. All children performed normally in the six-minute walking test. LUS was normal in the majority of patients (73.3%); 11.9% had assigned a score of 1 and 14,9% had a score of 2. None of the children exhibited an LUS score of 3, suggesting areas of consolidation or ‘white lung’. In general, LUS was negative in 73.0% and positive in 27.0%. No notable correlation was observed between asthma, allergic rhinitis, atopic sensitization, and symptoms of COVID-19, duration of the acute disease or LUS score, and chest auscultation or lung functionality. (Table 1).

**Figure 2:**
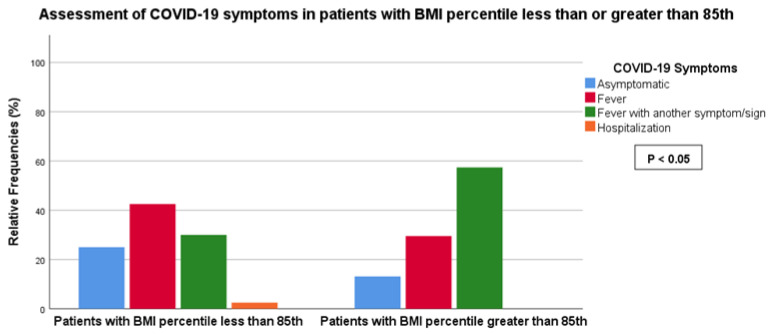
Association between LUS score and BMI less or greater than the 85th percentile.

**Figure 3:**
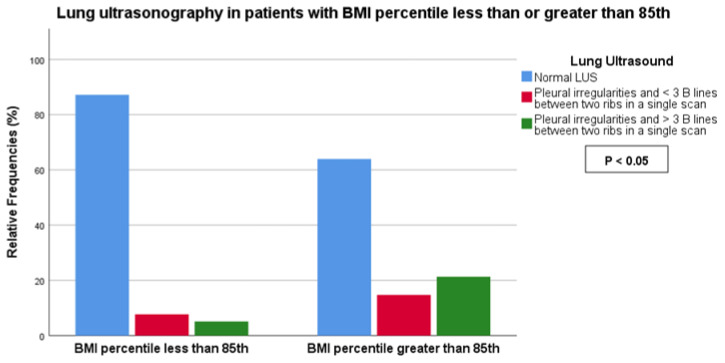
Association between COVID-19 symptoms and BMI less or greater than the 85th percentile.

We observed a statistically significant association between BMI (< vs. >85th percentile) and the symptomatic score of COVID-19 (score 0: 25.0% vs. 13.1%, score 1: 42.5% vs. 29.5%, score 2: 30.0% vs. 57.4%, score 3: 2.5% vs. 0%, *p*-value < 0.05) as well as LUS score (score 0: 87.2% vs. 63.9%, score 1: 7.7% vs. 14.8%, score 2: 5.1% vs. 21.3%, *p*-value < 0.05) (Figure 2 and Figure 3). No association was found between BMI and the duration of COVID-19.

## 4. Discussion

In our study, we showed that being overweight (BMI > 85th percentile) is associated with LUS abnormalities and COVID-19 symptoms in children with long COVID respiratory manifestations. Neither asthma nor atopy were found to be correlated with a major risk of severe illness or lung alterations. The association between BMI in acute COVID-19 disease and post-COVID-19 pathology has already been highlighted in the literature, demonstrating elevated rates of severe illness, hospitalization, and mortality in overweight and obese children in comparison to those with a normal BMI [63,64,65,66]. The association between BMI and long COVID may be ascribed to various factors. Individuals with a high BMI may have altered immune systems and chronic inflammatory responses, influencing the management of COVID-19 infection and the resolution of post-acute inflammation. Obesity itself is linked to reduced lung function and increased susceptibility to respiratory complications, contributing to the severity of respiratory symptoms in long COVID. Moreover, obesity-related comorbidities, such as diabetes and heart diseases, can exacerbate the persistence of symptoms.

The amount of research that has investigated the relationship between comorbidities including allergic diseases, long COVID, and LUS findings in children is limited.

In the Italian research by Mancino et al., a relatively large portion of long COVID children, reporting persisting symptoms, was associated with symptom burden and atopy (*p*-value = 0.006). The number of overweight and obese females was statistically greater in symptomatic patients compared to asymptomatic ones (*p* = 0.03) [67]. Also, regarding the identification of LUS abnormalities, our results align with those reported in the literature. In the study conducted by La Regina et al., prevalent findings included irregular pleural lines in about 30%, followed by B-lines in about 17%, and subpleural consolidations in about 9% of the cases. Furthermore, this study also demonstrated a positive correlation between BMI and the occurrence of B-lines in LUS (*p* < 0.001) [60]. In the article by Cantinotti et al., children who had had a symptomatic SARS-CoV-2 infection showed statistically higher LUS scores than asymptomatic ones [68]. 

On the other hand, in the prospective observational case–control study by Gräger et al., LUS findings did not differ between 30 children with long COVID and 15 lung-healthy children. Furthermore, there was no significant correlation between LUS findings and clinical data [69]. However, the size of the patient sample was quite limited. Another noteworthy discovery that surfaced in our study is that, in our sample, of the 27 patients who had a positive ultrasound, 90% showed a negativization in their LUS after 3 months, 9% after 6 months, and one patient after 9 months from the acute infection. In our previous study, we highlighted that COVID-19 LUS abnormalities may require time to fully dissipate, even with improvements in respiratory symptoms and lung functionality. Hence, it could be beneficial to persist in monitoring the pulmonary condition using LUS in the subsequent months. This approach allows the conservation of radiation-based investigations (such as X-rays and CT scans) for later stages or when deemed necessary [70]. Understanding the implications of LUS picture negativization over time could provide valuable insights into the natural course of the disease, potential recovery mechanisms, and the resilience of the pediatric respiratory system. It may also inform strategies for monitoring and managing post-COVID-19 lung conditions in this specific demographic, contributing to improved healthcare practices and patient outcomes. In the study conducted by Denina et al. in Italy, it was observed that out of thirteen children who exhibited a pathological LUS during their hospitalization for COVID-19, a follow-up conducted 35 days after discharge revealed that three patients still displayed a mild interstitial pattern, and two cases exhibited multiple subpleural consolidations. A month later, one of the individuals underwent a subsequent examination, which showed complete normalization of the LUS. However, the other individual, who had cystic fibrosis, exhibited results likely associated with their pre-existing chronic lung condition [71]. In Zubairi et al.’s follow-up study on COVID-19, the majority of patients experienced complete normalization of their LUS within 5 weeks post hospital discharge. It is worth noting that their study had a limited patient population [72]. In the research by Buonsenso et al., regarding pediatric COVID-19 follow-up, within three months of the acute infection, about 39% of patients showed pleural line irregularities, and about 15% exhibited B-lines, compared to 10% and 8% after nine months. Almost all children displayed either a normal LUS pattern or minimal artifacts, signifying complete lung recovery post initial infection. Importantly, the LUS findings were similar in both the recovered children and those who developed long COVID [61].

The evolving landscape of medical research and technology opens up potential applications of artificial intelligence (AI) in the assessment and monitoring of pediatric patients with long COVID. Integrating AI into these processes holds the promise of enhancing diagnostic accuracy and offering valuable insights into the long-term respiratory effects [73,74,75].

Our study has some limitations, including the design being limited to a single center, the absence of a control group (children without a history of COVID-19 or those without long COVID), the lack of LUS scans performed during acute infection or preceding SARS-CoV-2 infection, as we cannot rule out the possibility that certain features were already present, the high prevalence of overweight in children in Naples, and the fact that we enrolled children with long COVID based solely on the persistence of respiratory symptoms.

## 5. Conclusions

Our research on respiratory long COVID and comorbidities in children provides valuable insights into the prolonged impact of the SARS-CoV-2 illness on respiratory health and its association with underlying medical conditions, in particular, overweight. The extended duration of respiratory symptoms beyond the acute phase of COVID-19 emphasizes the need for comprehensive post-acute care and ongoing monitoring for individuals with persistent respiratory issues. Taking action to reduce weight, encourage physical activity, and promote a balanced diet may help alleviate complications and sequelae from COVID-19. Improved understanding of these aspects will contribute to the development of targeted therapies and personalized interventions, ultimately enhancing the quality of life for children grappling with lingering respiratory symptoms post-COVID-19. Additionally, we think that lung ultrasound should become a part of the routine monitoring of respiratory infections with persistent sequelae, such as COVID-19. This simple and non-invasive method can offer valuable insights for monitoring, presenting an alternative before resorting to initial radiological investigations.

## Figures and Tables

**Table 1:** Baseline characteristics of the population.

Baseline Characteristics of the Population	
Male	64.7%
Female	35.3%
Mean age	8.92 years
BMI: -<75° -75–85° ->85°	41.6% 20.8% 37.6%
COVID-19 disease: -asymptomatic -fever -fever and respiratory symptoms -hospitalization with O_2_ supplementation	17.6% 35.3% 46.1 1%
Duration of COVID-19: ->15 days -<15 days	55.% 44.1%
Comorbidities: -allergic rhinitis, -46.1% asthma and -56.9% reported an allergic sensitization	28.9% 46.1% 56.9%
Spirometry: -FEV1/FVC > 80% -FEV1/FVC < 80%	98% 2%
Six-minute walking test: -Negative -Positive	100% 0%
LUS score: -score 0 -score 1 -score 2 -score 3	73.3% 11.9% 14.9% 0%

## Data Availability

The data sets used in the current study may be available from the corresponding author on reasonable request. The data are not publicly available due to privacy reasons.

## Supplementary Materials

The following supporting information can be downloaded at: https://www.mdpi.com/article/10.3390/children11020166/s1, File S1: follow-up COVID-19 patient’s card.
