# Supplementary material for: COVID-19 Pediatric Follow-Up: Respiratory Long COVID-Associated Comorbidities and Lung Ultrasound Alterations in a Cohort of Italian Children
Source: Children (Basel). 2024 Jan 27;11(2):166. doi: 10.3390/children11020166 (PMC10887372; doi:10.3390/children11020166)
Supplement: Supplementary file 1 [file children-11-00166-s001.zip › children-2815235-supplementary.pdf]

SCHEDA VALUTAZIONE PAZIENTE IN FOLLOW-UP PER COVID-19

NOME …………………… COGNOME ……………………

DATA DI NASCITA ……………………..

DATA PRIMO TAMPONE POSITIVO ………………… DATA TAMPONE NEGATIVO ………………..

DURATA MALATTIA …………………..

GESTIONE MALATTIA: □ A DOMICILIO □ IN OSPEDALE

SINTOMATOLOGIA:

- FEBBRE (T max ………. DURATA…………)
- TOSSE
- DOLORE PLEURICO
- FAME D'ARIA
- $SpO_2 < 95\%$
- ASINTOMATICO
- ALTRO ……………………………………………………………………

TERAPIA PRATICATA: DURATA TERAPIA ……………………….

- ANTIPIRETICO
- CORTISONE
- ANTIBIOTICO
- OSSIGENO
- ALTRO…………………………………………………………………..

IL PAZIENTE ERA AFFETTO DA QUALCHE PATOLOGIA RESPIRATORIA IN PRECEDENZA? □ SI □ NO

Se sì specificare quale e se ci sono state esacerbazioni della malattia:

……
……
………..

ESAME OBIETTIVO TORACICO:

……
……

SIX MINUTE WALKING TEST:

SPIROMETRIA BASALE …………………………………………………………………………………….

SPIROMETRIA DOPO TEST DI BRONCODILATAZIONE CON SALBUTAMOLO …………………………………………….

ECOGRAFIA POLMONARE:

……
……
……
……
……
